# *Strongyloides stercoralis* hyperinfection presenting with shock and intermittent eosinophilia: A case report

**DOI:** 10.1097/MD.0000000000030490

**Published:** 2022-09-02

**Authors:** Jih Tze Tan, Chih-Wei Tseng

**Affiliations:** a School of Medicine, Tzu Chi University, Hualien, Taiwan (R.O.C.); b Division of Gastroenterology, Department of Medicine, Dalin Tzu Chi Hospital, Buddhist Tzu Chi Medical Foundation, Chia-Yi, Taiwan (R.O.C.).

**Keywords:** eosinophilia, hypoalbuminemia, parasitic infection, shock, *Strongyloides stercoralis*

## Abstract

Strongyloidiasis is a parasitic disease caused by *Strongyloides stercoralis*. The clinical presentation varies according to the stage of infection. Diagnosing strongyloidiasis is a challenge in clinical practice due to the inconsistency of eosinophilia and the low sensitivity of standard microscopic stool examination. Strongyloides infection presenting with shock is rare.

**Patient concerns:**

In this case, the condition of a 77-year-old immunocompromised patient with intermittent diarrhea progressed to shock and hypoalbuminemia. Reviewing her medical records, we learned that she had experienced intermittent peripheral eosinophilia during the past 10 months. Although a series of examinations were done, the disease progressed and the diagnosis remained uncertain.

**Diagnosis:**

Using standard microscopic stool examination and gastroduodenscopy with biopsy, a diagnosis of strongyloidiasis was made.

**Interventions:**

After the diagnosis of strongyloidiasis was made, 2 courses of ivermectin were administered.

**Outcomes:**

The patient recovered uneventfully after treatment and there is no recurrence of eosinophilia in 1 year follow-up.

**Lessons:**

This report provides a brief review of the current modalities used for diagnosing strongyloidiasis. It emphasizes the low sensitivity of microscopic examination, and highlights the role of gastroduodenoscopy in the diagnosis of strongyloidiasis. This report also assures that patients with strongyloidiasis have a good prognosis when they are treated timely and appropriately.

## 1. Introduction

Strongyloidiasis is a parasitic disease caused by infection with *Strongyloides stercoralis*. The clinical manifestations of *S. stercoralis* infection vary according to the stage of infection and the immune condition of the host. In more than 60% of cases, an infected host may be asymptomatic or may have dermatologic, pulmonary, or gastrointestinal (GI) symptoms.^[1]^ In the immunocompromised host, strongyloidiasis infection can lead to hyperinfection or disseminated infection, both of which are associated with high mortality rates.^[2]^ The varied clinical presentations, inconsistency of eosinophilia, and the low sensitivity of standard microscopic stool examination delays the diagnosis, and can lead to misdiagnosis in nonendemic areas.

Here we report the case of a patient with strongyloidiasis who had intermittent diarrhea and hypereosinophilia. She was first seen in the outpatient department, where the diagnosis was inconclusive. The infection progressed to hyperinfection with shock, conditions that can mimic other bacterial infections.

The aim of this report is to address the challenges and limitations involved in diagnosing Strongyloides infection, and to raise awareness of this infection in non-endemic areas.

## 2. Case presentation

A 77-year-old woman with a history of end-stage renal disease was admitted to our hospital with abdominal pain and hypotension that had developed during a regular hemodialysis session. During the past week the patient had experienced intermittent abdominal pain, diarrhea, and postprandial vomiting. Upon arrival at the hospital, her heart rate was 91 beats per minute, blood pressure was 85/53 mm Hg, body temperature was 36°C, and respiratory rate was 19 breaths/min.

On physical examination, the patient appeared chronically ill and cachectic. Her abdomen was soft without local tenderness, while her lungs, heart, and soft tissue were normal. Laboratory studies revealed leukocytosis, 9990/µL; neutrophils, 66%; and eosinophils, 16.2%. In addition, she had normocytic anemia (hemoglobin, 7.1 g/dL; mean corpuscular volume, 90.2 fL; and hypoalbuminemia, 1.01 g/dL). Volume resuscitation and administration of vasopressor and empirical antibiotics, including the antiparasitic agent ceftriaxone, were administered to treat the tentative diagnosis of septic shock.

A review of her past medical records showed that the patient had had intermittent peripheral eosinophilia with increased total immunoglobulin E greater than 5000 IU/mL about 10 months before. Results of a series of tests, including microscopic stool examinations and a review of autoimmune markers, including antinuclear antibody, complement C3, complement C4, myeloperoxidase antibody, and cytoplasmic anti-neutrophil cytoplasmic antibodies, were all normal. The peripheral eosinophilia improved with systemic steroid administration. However, she had a history of intermittent abdominal fullness, nausea, vomiting, diarrhea, and hypotension during the past 10 months.

**Figure 1.**
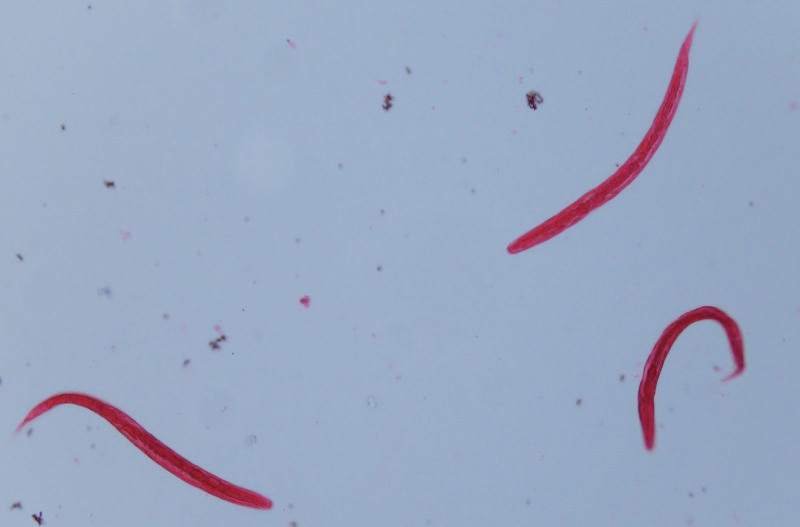
The larva of *Strongyloides stercoralis* in feces (MIF stain, ×200).

**Figure 2.**
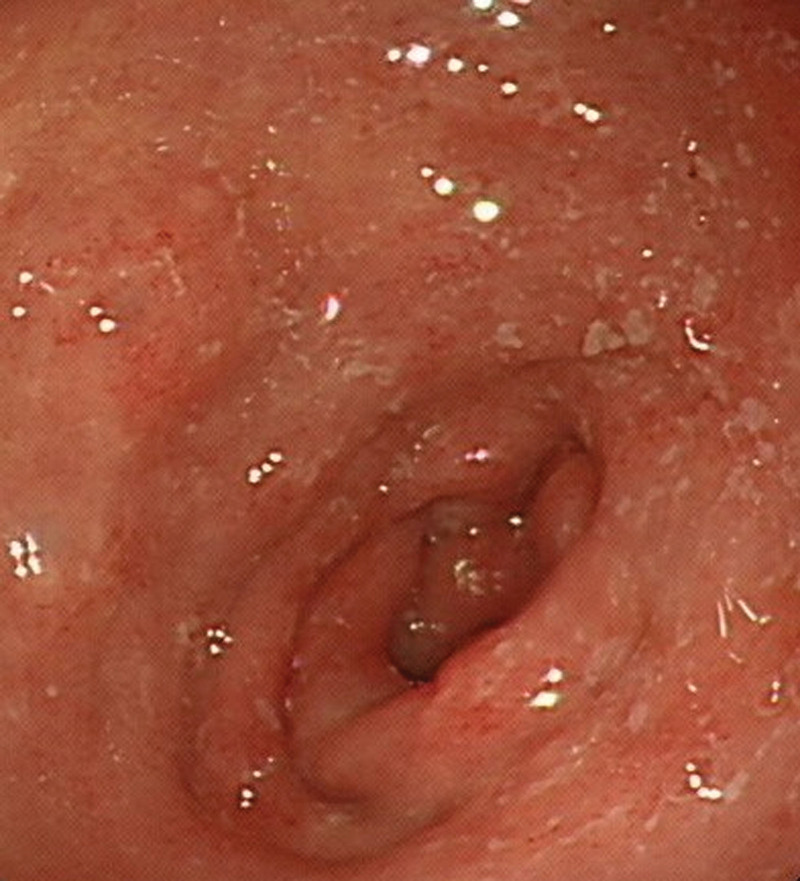
Gastroduodenoscopy showed duodenitis with a whitish mottled surface.

**Figure 3.**
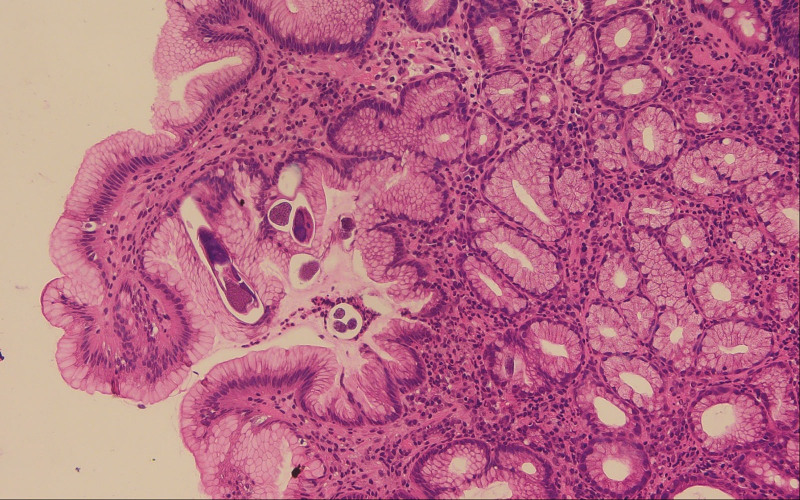
Nematode-like material (see black arrow at lower left) is surrounded by neutrophils and eosinophils within the glandular lumens and glandular epithelial walls (Hematoxylin and eosin stain, ×100).

After admission, several episodes of fever (body temperature > 38°C), tachycardia (heart rate > 90 beat/min) and mental status changed (Glasgow Coma Scale 13-14) were reported. All these findings in association with abdominal pain and diarrhea further supported the diagnosis of septic shock. Persistent diarrhea (8 times per day) and eosinophilia (2991/µL) were reported too. Five sets of stool specimens were checked, and the last 2 sets revealed the presence of *S. stercoralis* (Fig. 1; Supplementary Video, Supplemental Digital Content 1, http://links.lww.com/MD/H248). The gastroduodenoscopy for anemia survey showed duodenal mucosal erosions with a whitish mottled surface (Fig. 2) and gastric ulcer. The histopathology report showed nematode-like material within the glandular lumens and glandular epithelial walls, surrounded by neutrophils and eosinophils (Fig. 3).

**Figure 4.**
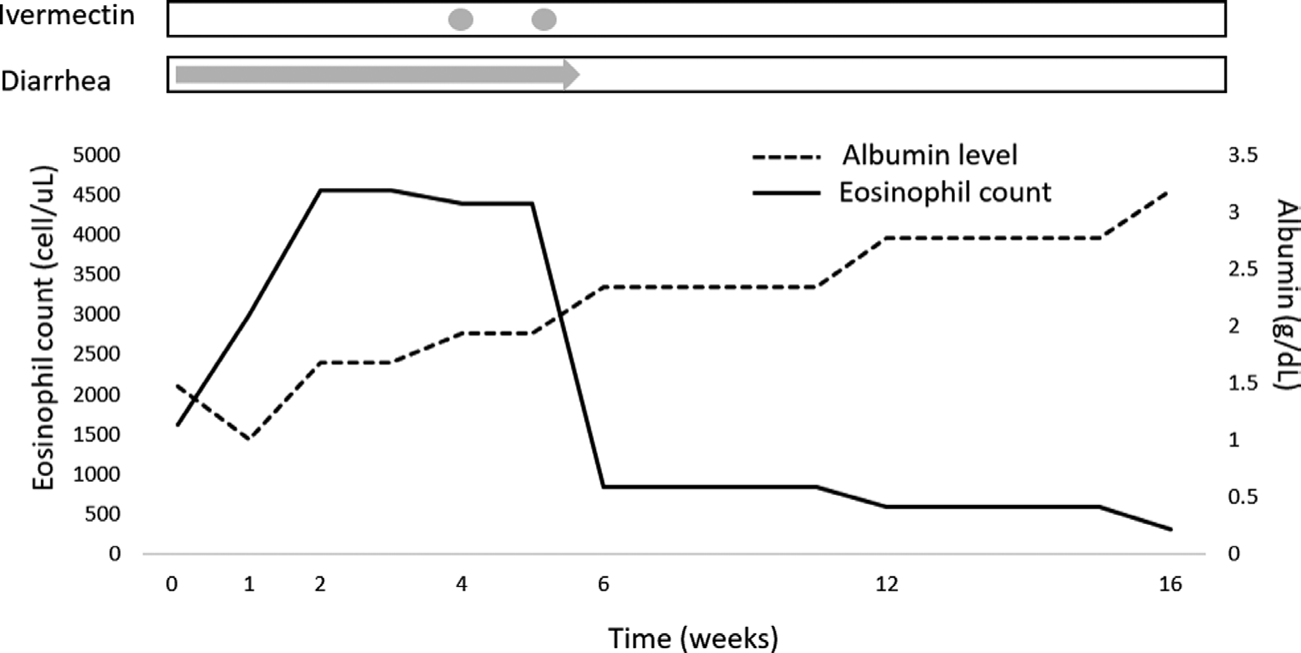
Charting the clinical course of the patient. Administration of medication correlated with serum albumin levels, eosinophil counts, and the clinical syndrome (diarrhea).

After the diagnosis of strongyloidiasis was made, 12 mg of ivermectin was administered consecutively for 2 days. A second course of ivermectin was prescribed again 1 week later because the patient still had diarrhea in spite of having 6 sets of normal stool specimens after the first course of ivermectin. The patient’s diarrhea and hypotension improved following 2 more courses of ivermectin treatment. The peripheral eosinophilia count returned to normal 1 month later and remained normal for 1 year. The albumin level gradually increased from 1.01 to 3.19 g/dL within 4 months (Fig. 4).

## 3. Discussion

Making a definitive diagnosis of Strongyloides infection involves visualization of the larvae by stool exam or through histopathologic findings. Strongyloidiasis is difficult to diagnose by the stool specimens alone if the parasitic load is low, and the larval output is irregular in most patients. Microscopic examination of a stool sample detects the parasite in 25% of cases. Multiple stool microscopic sample examinations may increase the sensitivity to 85%.^[3]^ In this case, we detected the parasite in the fourth stool sample (Fig. 1). In addition to collection of consecutive stool samples, using concentrations and parasitologic methods can increase the diagnostic rate. Among those parasitologic detection methods, agar plate culture (sensitivity: 89%) has the highest efficacy for diagnosis of strongyloidiasis, followed by the Baermann concentration and formalin ethyl acetate methods.^[4,5]^

Some serum examinations, such as for Strongyloides antibodies, and polymerase chain reaction (PCR) tests, were added to enhance the diagnosis. ELISA tests could detect the serum IgG against filariform larvae of *S. Stercoralis* but the sensitivity and specificity were inconsistent (sensitivity 88%–95%, specificity 29–99%).^[6–8]^ Strongyloides infection also has cross-activity with other parasitic infections; thus, the presence of antibody does not distinguish between current and past infections.^[8]^ The PCR has low sensitivity (56–71%) and high specificity (93%–95%).^[9,10]^ However, due to the unavailability of PCR tests, microscopic examination remains the main diagnostic method in most hospital settings.

This case highlights the importance of performing an endoscopic study with mucosal biopsy for detection of GI strongyloidiasis (Figs. 2 and 3). Although strongyloidiasis has a broad range of endoscopic features, the most frequent findings on endoscopy include ulcerations, gastritis, or duodenitis. Multiple biopsy specimens could yield the diagnosis in 90% of cases.^[11,12]^ Histopathologic findings may reveal *S. stercoralis* larvae, eggs, or adult forms located in the gastric and/or duodenal crypts. Eosinophils infiltrating the lamina propria might be found, and the intensity can be correlated with the intensity of the infection.^[13]^

The phenomenon of intermittent eosinophilia in this case was related to the auto-infective cycle of *S. stercoralis*. Eosinophilia is notable only when the immune regulatory cells react to the parasite or its products. Therefore, not all parasitic infections will demonstrate persistent eosinophilia, especially those limited to intraluminal infection.^[14]^ In *S. stercoralis* infections, eosinophilia may be detected during migration of larvae through perianal skin or intestinal mucosa. Furthermore, the host response to parasitic infection is determined by the host’s immune response and the medications being given. Some drugs, especially glucocorticoids, can induce eosinophil apoptosis, resulting in a decreased eosinophil count.^[15]^ The inconsistency of eosinophilia and low sensitivity of a standard microscopic stool examination make strongyloidiasis a disease that is frequently misdiagnosed.^[16,17]^ Therefore, detecting Strongyloides infection requires a high level of suspicion in spite of multiple negative microscopic examinations. We demonstrated that the eosinophil count will normalize within 1 month after treatment is begun (Fig. 4). The delayed normalization of the eosinophil count is probably due to an immune response toward dead parasites that remain in the organs.

Protein-losing gastroenteropathy was the likely cause of the hypoalbuminemia status in this patient. Strongyloides has been documented to cause protein-losing gastroenteropathy and malabsorption,^[18,19]^ due to the inflammatory changes that involve the gastric crypt, duodenum, and small intestine.^[18–21]^ Protein-losing gastroenteropathy caused by parasites is reversible if treated optimally. In this case, hypoalbuminemia improved to near-normal levels within 4 months after Strongyloides was eradicated.

## 4. Conclusion

Diagnosing strongyloidiasis is challenging due to the low sensitivity of microscopic examination and the inconsistency of eosinophilia. Multiple stool samplings may increase the sensitivity to 85%. In this case, endoscopic study with mucosal biopsy specimens was the most sensitive approach for diagnosing GI strongyloidiasis. The eosinophilia count and protein-losing gastroenteropathy were reversible after the parasite was eradicated.

## Acknowledgments

The authors thank the patient, who consented to having her data published in this case report.

## Author contributions

JTT provided material support and drafting of the manuscript. C-WT provided material support and critical revision of the manuscript for important intellectual content.

**Conceptualization:** Jih Tze Tan, Chih-Wei Tseng.

**Data curation:** Jih Tze Tan, Chih-Wei Tseng.

**Formal analysis:** Jih Tze Tan, Chih-Wei Tseng.

**Methodology:** Chih-Wei Tseng.

**Project administration:** Chih-Wei Tseng.

**Resources:** Chih-Wei Tseng.

**Supervision:** Chih-Wei Tseng.

**Validation:** Chih-Wei Tseng.

**Visualization:** Chih-Wei Tseng.

**Writing – original draft:** Jih Tze Tan, Chih-Wei Tseng.

**Writing – review & editing:** Jih Tze Tan, Chih-Wei Tseng.

## Supplementary Material


